# Impact of Frailty and Other Factors as Estimated by HU to Predict Response to Anabolic Bone Medications †

**DOI:** 10.3390/jcm14093247

**Published:** 2025-05-07

**Authors:** Abdelrahman M. Hamouda, Zach Pennington, Rahul Kumar, Michael L. Martini, Derrick Obiri-Yeboah, Maria Astudillo Potes, Nicholas Kendall, Anthony L. Mikula, Michelle J. Clarke, William E. Krauss, Ahmad N. Nassr, Brett A. Freedman, Arjun S. Sebastian, Melvin D. Helgeson, Kurt A. Kennel, Jeremy L. Fogelson, Benjamin D. Elder

**Affiliations:** 1 Departement of Neurologic Surgery, Mayo Clinic, 200 1st Street SW, Rochester, MN 55905, USAkendall.nicholas@mayo.edu (N.K.);; 2 Departement of Orthopedic Surgery, Mayo Clinic, 200 1st Street SW, Rochester, MN 55905, USA; 3 Division of Endocrinology, Mayo Clinic, 200 1st Street SW, Rochester, MN 55905, USA

**Keywords:** osteoporosis, spine surgery, Hounsfield units, anabolic therapy, antiresorptive therapy, frailty, bone mineral density, vertebral bone quality

## Abstract

**Introduction:** Bone health optimization is a key component of the preoperative management of spine surgery patients, as poor bone quality increases the odds of mechanical complications. The present study aimed to achieve the following: (1) compare the relative efficacy of current osteoporosis medications in improving bone quality; (2) identify factors influencing treatment response in preoperative spine surgery patients. **Methods:** Patients treated at a single, multisite institution who received osteoporosis treatment were identified. Data were gathered on pre- and post-treatment lumbar spine Hounsfield Unit (HU) measurements, patient demographics, frailty scores (modified Frailty Index/mFI, risk analysis index/RAI), and pharmacologic treatment details. The primary outcome was a ≥7 point improvement in lumbar HU, and baseline and logistic regression models were utilized to identify factors associated with this improvement. Medications were grouped as anabolic (teriparatide, romosozumab) and antiresorptive (denosumab, alendronate) therapies. **Results:** A total of 267 patients were included (median age: 74 years; IQR [66–81]; 67.3% female), with 127 (47.6%) improving by ≥7 HU. The treatment agents used were alendronate (95), denosumab (113), romosozumab (31), and teriparatide (28). Univariable comparisons revealed significant differences across medication groups in age (*p* < 0.001), sex (*p* < 0.001), mFI (*p* < 0.001), RAI (*p* = 0.004), BMI (*p* < 0.001), pre-treatment HU (*p* = 0.022), and treatment duration (*p* < 0.001). The highest HU improvement rates (ΔHU ≥ 7) were observed in patients receiving the anabolic medications romosozumab (67.7%) and teriparatide (60.7%). Univariable logistic regression identified male sex (OR 0.54, *p* = 0.019), higher pre-treatment HU (OR 0.99, *p* = 0.006), and longer treatment duration (OR 0.97, *p* = 0.003) as factors associated with lower odds of HU improvement. Only romosozumab was associated with significantly higher odds of HU improvement relative to alendronate (OR 3.02, *p* = 0.012). In our multivariable analysis, male sex (OR 0.53, *p* = 0.028) and higher pre-treatment HU (OR 0.99, *p* = 0.002) remained significant predictors of HU improvement. However, medication type was not significant in the multivariable analysis. **Conclusions:** Our study highlights that male sex and higher pre-treatment HU were independently associated with lower odds of HU improvement, while medication type was not a significant predictor. Additionally, anabolic agents offered superior improvement relative to antiresorptive therapies.

## 1. Introduction

Osteoporosis—characterized by low bone mineral density (BMD) and associated microarchitectural deterioration resulting in poor bone strength—affects approximately 10 million patients in the United States and can lead to fragility fractures and long-term disability [1]. Among spine surgery candidate patients, poor bone strength portends an increased risk of mechanical complications and pseudarthrosis following instrumented fusion. This includes increased odds of implant failure (e.g., screw loosening or interbody graft subsidence), vertebral fractures, and proximal junctional kyphosis (PJK) [2,3,4,5]. Consequently, bone health optimization has become a key component of the preoperative optimization of spine surgery patients [6,7].

For patients with low BMD, pharmacologic intervention is generally considered the treatment of choice. Osteoporosis medications can be grossly classified into antiresorptive and anabolic medications. The former (e.g., bisphosphonates, denosumab) maintain or modestly increase BMD by prohibiting bone resorption; however, many surgeons may avoid these agents as they can interfere with the normal bone maturation process and potentially increase the risk of pseudoarthrosis. In contrast, the latter (romosozumab, abaloparatide, and teriparatide) improve BMD and bony microarchitecture by stimulating bone formation [8]. Additional risk factors may modify the real-world effectiveness of these medications, including smoking, physical activity level, dietary habits (e.g., calcium and vitamin D intake), glucocorticoid use, medication non-compliance, and patient frailty [9,10]. In general, anabolic agents are preferred for patients with very low BMD, as they have been demonstrated to significantly improve BMD. Within the class of anabolic agents, romosozumab—a sclerostin inhibitor—has been suggested to be more effective at improving BMD [6,11].

The effectiveness of anti-osteoporosis medications has conventionally been assessed radiographically with dual-energy X-ray absorptiometry (DEXA). However, DEXA cannot distinguish improvement in bone strength due to increased bone volume and mass as a result of anabolic therapy from the increased mineralization of existing bone due to antiresorptive therapy. Moreover, DEXA is commonly inaccurate in patients with degenerative spinal pathologies [12]. Opportunistic measures, such as computed tomography (CT)-based Hounsfield units (HU) and MR-based vertebral bone quality (VBQ) scores, appear less subject to these changes and have become increasingly popular [12]. Not only do these metrics spare patients additional testing, but they also directly assess the bones undergoing instrumentation. CT-based HU have been strongly correlated with biomechanical CT estimates of bone strength, which can assay and track both bone density and microarchitectural changes [5,6,13,14].

A remaining question is the extent to which factors, such as sex, age, and frailty affect patient response to anti-osteoporosis therapy. The present study sought to evaluate the impact of patient demographics and comorbidities on response to anti-osteoporosis therapy, as measured by changes in HU.

## 2. Methods

### 2.1. Data Source

In this retrospective study, and following IRB approval from the Mayo Clinic Institutional Review Board (IRB #18-002622), patients treated for osteoporosis at a single, multisite institution were identified. Patients were included if they underwent treatment for osteoporosis between 2005 and 2022, were >18 years old at the time of treatment, and had undergone one of the following anti-osteoporosis therapies: alendronate or denosumab for ≥1 year, or romosozumab or teriparatide for ≥3 months. Due to the common use of alendronate and teriparatide at our institution, these medications were selected out of their respective classes for the analysis. Eligible patients were required to have pre-treatment and post-treatment non-contrast CT imaging obtained within 1 year prior to and following the completion of therapy (Figure S1). For patients treated with alendronate, a convenience sample was selected by reviewing cases in reverse chronological order. For the other drug groups, all eligible patients within the study period were included due to smaller sample sizes. Patients were excluded if they had a history of vertebroplasty or instrumentation involving the L1–L4 levels. Patients were also excluded if they had undergone contrast-enhanced CT or if their imaging demonstrated evidence of extensive sclerosis, lytic lesions tumors, infections, or fractures. The primary outcome of interest was improvement of ≥7 HU from pre- to post-treatment CT scan, as previous estimates of the measurement error for HU have been reported as 7 units [15].

The records of patients meeting the inclusion/exclusion criteria were queried for data on demographics (age, sex, body mass index/BMI), smoking status, anti-osteoporosis treatment (agent, duration of treatment), frailty, and pre-and post-treatment bone density based on HU. Frailty was assessed using the 5-item modified Frailty Index (mFI-5) and the risk analysis index (RAI). Bone density was assessed using Hounsfield units (HU) on pre- and post-treatment non-contrast CT scans of the lumbar spine. CT scans were all obtained within 1 year of treatment initiation and completion. HU values were measured as previously described, with regions of interest drawn on axial CT slices through the L1–4 vertebrae. Three ROIs were drawn for each vertebra, with one immediately inferior to the cranial endplate, one through the mid-body, and one immediately superior to the caudal endplate [4,5,16]. The HU values were then averaged across all 12 ROIs.

### 2.2. Statistical Analysis

Data were collected using Microsoft Excel (Redmond, WA, USA) and analyses were performed using R (v. 4.3.1) and R Studio (v. 2024.04.1). The primary outcome of interest was a ≥7 point improvement in average HU from pre- to post-treatment CT. Continuous variables were summarized as medians with interquartile ranges (IQRs), and categorical variables were presented as counts and percentages. Univariable non-time-adjusted analyses were conducted using the Kruskal–Wallis test for continuous variables and χ^2^ tests for categorical and dichotomous outcomes. To assess factors associated with HU improvement (Δ ≥ 7 HU), univariable logistic regression was performed. To estimate the impact of risk factors and predict ΔHU, univariable linear regression was conducted. Backwards selection was utilized for the construction of the final multivariable models using Akaike information criteria (AIC) minimization. Time was included as a covariate in the multivariate models to account for the differences in treatment duration inherent to each medication class. This adjustment aimed to control for the potential effects of varying exposure times on outcomes.

## 3. Results

**Figure 1:**
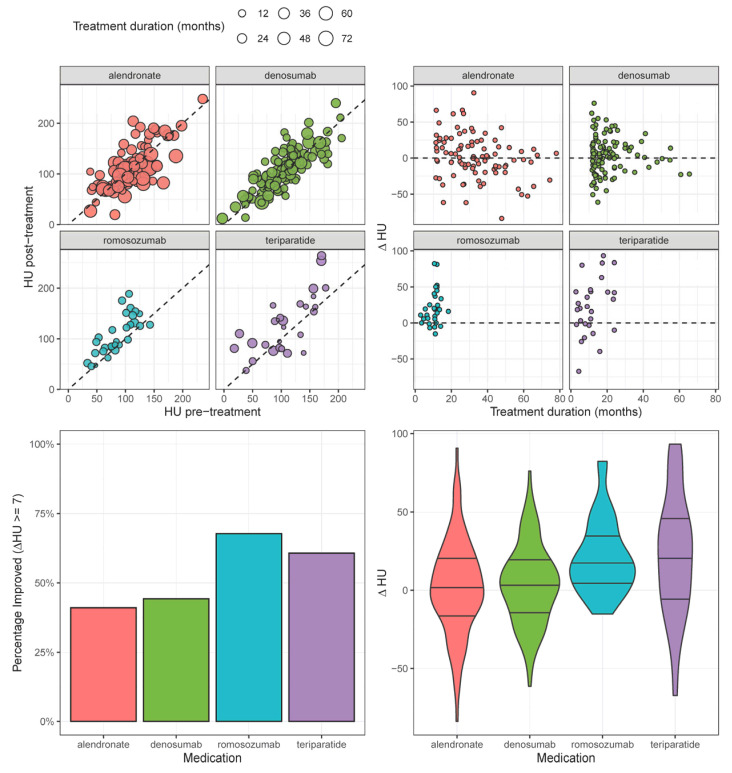
HU change before and after treatment, stratified by medication type.

A total of 267 patients were included (median age: 74 years; IQR [66–81]; 67.3% female), with 127 (47.6%) improving by ≥7 HU. The anti-osteoporosis treatments employed were as follows: alendronate (n = 95), denosumab (n = 113), romosozumab (n = 31), and teriparatide (n = 28). Univariable comparisons revealed significant differences across medication groups in age (*p* < 0.001), sex makeup (*p* < 0.001), mFI score (*p* < 0.001), RAI score (*p* = 0.004), BMI (*p* < 0.001), pre-treatment HU (*p* = 0.022), and treatment duration (*p* < 0.001). Smoking history did not significantly differ between groups. The rates of HU improvement (≥ 7 HU), in decreasing order, were as follows: romosozumab (67.7%), teriparatide (60.7%), alendronate (41.1%), and denosumab (26.5%). The median ΔHU values, in decreasing order, were as follows: teriparatide (22.1 [−2.46–22.5]), romosozumab (18.3 [5.5, 22.5]), denosumab (3.3 [−12.6, −22.5]), and alendronate (1.6 [−13.0, 22.5]) (Table 1) [Figure 1].

In our univariable analysis (Table 2), the odds of improvement (ΔHU ≥ 7) were negatively correlated with male sex (OR 0.54; 95% CI [0.32, 0.90]; *p* = 0.019) and higher baseline HU (0.99/unit; [0.98, 1.00]; *p* = 0.006). Among the medications, romosozumab was significantly associated with higher odds of HU improvement (OR 3.02; [1.31, 7.35]; *p* = 0.012) relative to treatment with alendronate. In our multivariable analysis, male sex (OR 0.53; [0.29, 0.93]; *p* = 0.028), higher baseline HU (0.99/unit; [0.98, 1.00]; *p* = 0.002), and age (OR 0.97/year; [0.96, 1.00]; *p* = 0.049) remained significantly associated with lower odds of HU improvement. However, none of the medications remained significantly associated with HU improvement in the multivariable model.

**Figure 2:**
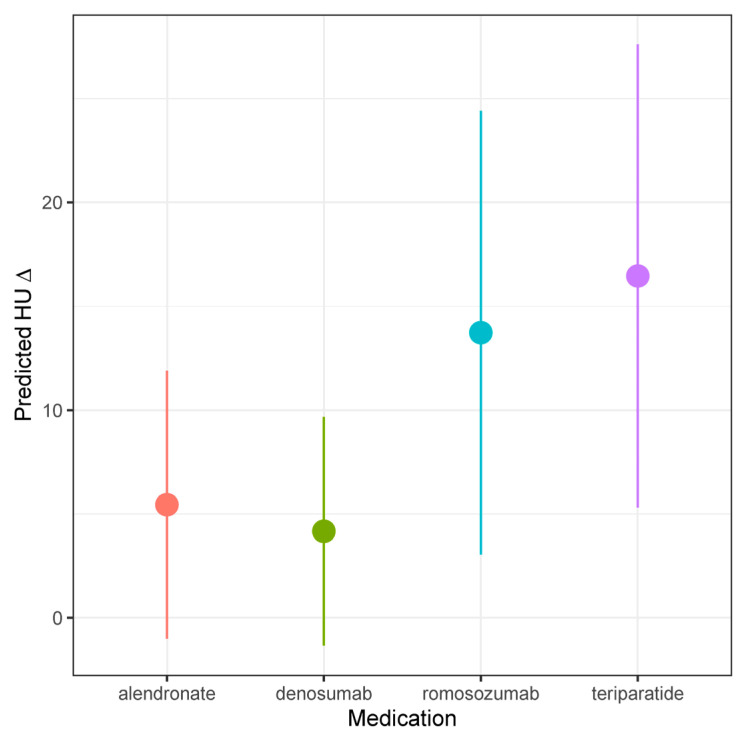
Predicted HU, stratified by medication type.

In our univariable linear regression analysis (Table 3) [Figure 2], ΔHU was negatively correlated with higher baseline HU (β −0.18/unit; [−0.2, −0.09]; *p* < 0.001). Romosozumab (β 19.39; [7.72, 31.05]; *p* = 0.001) and teriparatide (β 20.09; [7.96, 32.21]; *p* = 0.001) were associated with significantly larger increases in ΔHU relative to alendronate. In our multivariable analysis, only higher baseline HU (β −0.19/unit; [−0.28, −0.10]; *p* < 0.001) and age (β −0.39/unit; [−0.72, −0.07]; *p* < 0.005) predicted ΔHU; the pharmacologic treatment employed was not significantly associated with ΔHU in the multivariable model.

## 4. Discussion

In the present analysis, patient factors, specifically older age, male sex, treatment duration, and baseline bone health (as measured by pre-treatment HU) were associated with both lower odds and magnitude of improvement. Although higher baseline HU typically reflects greater bone mass and might be expected to correlate with better improvement, our findings suggest an inverse relationship and are consistent with those of Tanphiriyakun et al. [17]. This may be explained by the fact that, in individuals with lower baseline bone density, even a modest absolute gain translates into a relatively larger increase in HU measurements. This effect may be particularly pronounced in patients receiving anabolic treatment, as these agents stimulate bone formation, leading to greater relative HU changes compared to antiresorptive therapy. Here, we additionally noted treatment duration to be inversely correlated with HU improvement and the odds of improving by ≥7 HU, which seems counterintuitive. We speculate that this is likely artifactual and reflects the fact that anabolic agents are prescribed for shorter periods of time, as after correcting for treatment duration and other relevant covariates in multivariable modeling, specific medication was not a statistically significant independent predictor of change in HU [18]. Notably, romosozumab and teriparatide were again found to have the most rapid improvement in HU and the greatest odds of improvement [6,18].

### 4.1. Osteoporosis and Mechanical Complications of Spinal Fusion

Low BMD is prevalent in older people, especially among women. Given the advancing age of patients undergoing spinal fusion, low BMD is increasingly common amongst spine surgery patients, with 34.2% having osteoporosis and 43.5% having osteopenia, as assessed by DEXA [19]. Multiple studies have correlated low BMD with increased odds of mechanical complications, including screw pullout, screw cut out, and interbody subsidence [12,20,21,22,23,24]. Additionally, low BMD is associated with increased odds of pseudoarthrosis and PJK [20,21,22]. However, despite the high prevalence of osteoporosis amongst spine surgery patients and its known association with poor outcomes, it remains underdiagnosed and undertreated [22,25]. Perioperative treatment has been associated with reduced postoperative complications and improved fusion rates, though the agent employed appears to be important [25,26].

Bisphosphonates are a type of antiresorptive therapy, typically administered for 3–5 years, with good efficacy in decreasing fracture risk, though questionable utility in preoperative optimization for spine surgery [18,27]. While some series have suggested that bisphosphonates may improve fusion rates [27], a recent meta-analysis by Buerba and colleagues found that the addition of bisphosphonates had no appreciable effect on rates of arthrodesis or screw loosening following thoracolumbar fusion [26]. Consequently, for patients who require the preoperative optimization of bone health for spine surgery, these are a poor option.

Denosumab is a RANK ligand inhibitor and is also an antiresorptive therapy that reduces fracture risk when administered for 5 years. It is more effective at increasing BMD compared to bisphosphonates [28,29,30], and has shown some utility in spine surgery patients. Ryu and colleagues compared outcomes in patients undergoing PLIF treated with rhBMP-2 or rhBMP-2 and systemic denosumab therapy [31]. They noted significantly lower rates of osteolysis in denosumab-treated patients, though fusion rates were similar. Finite element analysis also suggested that denosumab treatment may improve bone strength and screw fixation. There was an approximately 10% increase in local volumetric bone mineral density at 24 months and a corresponding 12% increase in compressive strength and 16% change in screw pullout strength. Notably, there was significant correlation of vBMD with both compressive strength (r = 0.67) and pullout strength (r = 0.83) [32]. The co-administration of teriparatide and denosumab may also increase BMD and fusion rates among PLIF patients relative to teriparatide alone, though clinical evidence supporting denosumab use in the spine surgery population is otherwise wanting [33].

As suggested by the present analysis, anabolic agents appear more promising for improving outcomes in the spine surgery population. Parathyroid hormone (PTH) receptor agonists are the oldest member of this group and include teriparatide (PTH 1–34) and abaloparatide (PTH-related peptide 1–34); they are administered by daily subcutaneous injection for up to two years. The administration of PTH analogs shows a substantial increase in BMD and a resultant decrease in VFs compared to placebo (65%) or bisphosphonates (55%) [34]. The data in the spine fusion population are also promising, with Seki et al. showing improved arthrodesis rates and decreased rates of adjacent-level fracture relative to bisphosphonate therapy following instrumented fusion for adult spinal deformity (ASD) [35]. Ebata et al. similarly showed a significant increase in arthrodesis rates following single-level lumbar interbody fusion in patients treated with teriparatide versus placebo [36]. Yagi and colleagues contemporaneously showed a decrease in the rate of PJK among ASD patients treated with teriparatide versus placebo. They also noted improvements in fine bone structure at the vertebra cephalad to the proximal instrumented level [37]. The extant literature is heterogeneous, though, and based upon small studies. The clinical trial of Jespersen et al. showed no significant difference in mean fusion mass volume or overall fusion rates between those treated with teriparatide and placebo [38]. Further investigation is therefore merited, including research into the benefits of combining teriparatide therapy with other anti-osteoporosis therapy.

Romosozumab is an antibody to sclerostin injected subcutaneously monthly for 12 months. Sclerostin inhibition results in a rapid and substantial rise in bone formation and the concurrent suppression of bone resorption, producing large increases in BMD and a reduction in VFs [39,40,41]. Evidence supporting its use in the spine surgery population is limited; however, Sawada et al. found its use to decrease PJK risk in patients undergoing surgery for ASD or VF [42].

Notably, anabolic medications are significantly more expensive than traditional antiresorptive treatments, raising concerns about their cost-effectiveness. While anabolic agents provide greater BMD improvements, studies suggest that, when evaluating quality-adjusted life years, they may not always be more cost-effective than antiresorptive therapies [43,44]. This highlights the need for further research to determine whether the superior BMD gains associated with anabolic treatments translate into meaningful clinical outcomes, such as reduced fracture rates and improved surgical fusion success.

### 4.2. Frailty, Patient Risk Factors, and Osteoporosis Treatment

The present results suggest that a patient’s odds of bone health improvement following osteoporosis therapy are independent of underlying frailty; even frail patients in the present study had a positive response to therapy. While the effects of frailty on osteoporosis treatment have not been specifically assessed in the spinal fusion population, Tanchanok et al. studied 29,904 Medicare beneficiaries hospitalized for new fractures and reported that treatment with osteoporosis medications led to similar reductions in subsequent fracture in frail and non-frail patients [45]. They additionally noted osteoporosis treatment to be less common among the frail group, highlighting the importance of increasing awareness about bone health optimization in this population. Spine surgery patients represent another high-risk population given the altered biomechanics created by instrumentation along with the significant costs of surgical revision. Consequently, the present data can be seen as further arguing for aggressive bone health optimization in both frail and non-frail spine surgery patients.

Regarding sex, males are often underdiagnosed and undertreated for osteoporosis, yet the existing literature suggests that men exhibit a similar response to osteoporosis treatment in terms of bone density and bone remodeling when compared to postmenopausal women [46]. However, our study found that males had a lower likelihood of bone density improvement, as estimated by HU. This discrepancy may be primarily attributed to the lower percentage of males receiving anabolic medications compared to females, highlighting their reduced access to effective treatment. Additionally, the underlying etiologies of bone loss differ between men and postmenopausal women, which may further contribute to this finding. In the latter group, the sharp decline in estrogen during menopause is thought to be the major driver of bone loss. By contrast, bone loss in men is generally multifactorial and is influenced by aging and chronic comorbidities that would not be amenable to hormonal therapies, such as PTH analogs. This is supported by evidence that men tend to experience worse outcomes after fractures [47]. With respect to age, older patients were found to be less likely to experience bone density improvement, which may be related to age-associated comorbidities such as diabetes mellitus and the natural decline in anabolic steroid hormones. Notably, responses to osteoporosis medication stratified by age and sex warrant further investigation, especially in patients undergoing spine surgery [48].

## 5. Limitations

There are several limitations to the present study. It is retrospective in nature, which precludes us from establishing any causative linkage. We were also unable to match cohorts based on medication types or control for all potential modifiable risk factors, including medication compliance, concurrent calcium/vitamin D supplementation, and concurrent renal disease. Additionally, this study is limited by a relatively small sample size; the recruitment of all patients from a single institution also introduces the potential for selection bias. Another key limitation of our study is the exclusion of patients without an evaluable vertebra, which inherently excludes individuals with prior fragility fractures, who are at the greatest risk of mechanical complications following spinal fusion. Given that fragility fractures are strong predictors of reduced bone strength and worse outcomes [49], excluding these patients may introduce a selection bias by omitting patients who are likely more osteoporotic and frail. These patients often represent the highest-risk cohort, with poor bone quality and increased susceptibility to instrumentation failure or pseudarthrosis. Though this was necessary for accurate HU assessment, these limitations may reduce the generalizability of our findings. Future studies should aim to include high-risk individuals to better inform perioperative decision-making in this vulnerable group. Moreover, while our study focused on HU changes as a surrogate for bone quality improvement, we did not correlate HU improvement with improvements in surgical outcomes such as fusion rates, hardware failure, or pain relief. Future prospective studies should explore whether HU gains translate into meaningful improvements in fusion outcomes, reoperation rates, or patient-reported outcomes. Finally, differing treatment durations among medication groups complicate direct comparisons. Although our inclusion criteria were defined based on the prior efficacy literature, these discrepancies may have influenced the magnitude of our observed HU changes.

## 6. Conclusions

Anabolic medications were associated with a greater percentage of patient improvement compared to antiresorptive medications. Males are less likely to receive anabolic medications compared to females. Additionally, patient factors, specifically older age and higher baseline bone density, were associated with lower odds of vertebral bone density improvement following osteoporosis treatment. These findings suggest that treatment response is influenced more by patient-specific characteristics than the class of therapeutic agent. Future prospective, multicenter studies incorporating standardized treatment durations and correlating bone density changes with surgical outcomes) are needed to validate these findings and guide optimal osteoporosis management in surgical populations, particularly in high-risk patients.

## Figures and Tables

**Table 1:** Univariable comparison of patients based on medication type.

	[ALL] *N* = 267	Alendronate *N* = 95	Denosumab *N* = 113	Romosozumab *N* = 31	Teriparatide *N* = 28	*p*-Value
Age	74.0 [66.0, 81.0]	71.0 [65.0–77.5]	81.0 [74.0, 85.0]	71.0 [60.0–77.0]	65.0 [61.0, 72.0]	<0.001
Sex:						<0.001
Female	172 (64.4%)	43 (45.3%)	78 (69.0%)	28 (90.3%)	23 (82.1%)	
Male	95 (35.6%)	52 (54.7%)	35 (31.0%)	3 (9.68%)	5 (17.9%)	
mFI5:						<0.001
mFI-0	109 (40.8%)	25 (26.3%)	43 (38.1%)	31 (100%)	10 (35.7%)	
mFI-1	88 (33.0%)	39 (41.1%)	40 (35.4%)	0 (0.00%)	9 (32.1%)	
mFI-2+	70 (26.2%)	31 (32.6%)	30 (26.5%)	0 (0.00%)	9 (32.1%)	
RAI	20.0 [13.0, 28.0]	21.0 [13.5, −27.5]	22.0 [16.0, 31.0]	18.0 [9.50, 20.0]	17.5 [9.50, 26.5]	0.004
BMI	25.6 [22.1, 29.9]	27.9 [23.7, 32.0]	24.0 [21.1, 27.9]	26.0 [23.0, 27.5]	24.1 [21.2, 27.5]	<0.001
Active smoker	25 (9.36%)	10 (10.5%)	8 (7.08%)	6 (19.4%)	1 (3.57%)	0.165
HU pre-treatment	103 [81, 126]	106 [89, 134]	106 [79, 123]	84 [67, 108.0]	102 [86, 146]	0.022
HU post-treatment	109 [82, 137]	109 [85, 132]	108 [80, 136]	98 [82, 130]	129 [82, 164]	0.430
Treatment duration (months)	18.0 [12.3, 30.0]	31.7 [21.7, 44.2]	17.1 [12.9, 23.9]	10.9 [8.02, 12.0]	10.4 [5.7, 17.0]	<0.001
ΔHU	5.8 [−11.0, 22.5]	1.6 [−13.0, 22.5]	3.3 [−12.6, 22.5]	18.3 [5.5, 22.5]	21.5 [−2.5, 22.5]	<0.001
Improved (ΔHU ≥ 7)	127 (47.6%)	39 (41.1%)	50 (44.2%)	21 (67.7%)	17 (60.7%)	0.028

Abbreviations: mFI5 = modified 5-item frailty index; RAI = risk analysis index; BMI = body mass index; HU = Hounsfield units.

**Table 2:** Logistic regression models for HU improvement (HU ≥ 7) (univariate and multivariate logistic regression).

	Univariable		Multivariable	
Variable	OR (95% CI)	*p*-Value	OR (95% CI)	*p*-Value
Male (ref: female)	0.54 [0.32, 0.90]	0.019	0.53 [0.29, 0.93]	0.028
mFI-1 (ref: mFI-0)	0.80 [0.44, 1.45]	0.460		
mFI-2+ (ref: mFI-0)	0.75 [0.43, 1.32]	0.325		
Treatment duration (months)	0.97 [0.95, 0.99]	0.002	0.99 [0.98, 1.00]	0.002
Age	0.98 [0.96, 1.00]	0.071	0.97 [0.95, 1.00]	0.049
BMI	0.98 [0.94, 1.02]	0.295		
HU pre-treatment	0.99 [0.98, 1.00]	0.006	0.99 [0.98, 1.00]	0.002
RAI	1.00 [0.98, 1.02]	0.945		
Active smoker	1.02 [0.44, 2.34]	0.964		
Denosumab (ref: alendronate)	1.14 [0.66, 1.98]	0.643	0.81 [0.40, 1.63]	0.554
Teriparatide (ref: alendronate)	2.22 [0.95, 5.38]	0.070	0.88 [0.31, 2.52]	0.811
Romosozumab (ref: alendronate)	3.02 [1.31, 7.35]	0.012	0.93 [0.32, 2.77]	0.902

**Table 3:** Linear regression models for ΔHU (univariate and multivariate linear regression).

	Univariable		Multivariable	
Variable	β (95% CI)	*p*-Value	β (95% CI)	*p*-Value
Male (ref: female)	−7.17 [−14.57, 0.23]	0.057		
mFI-1 (ref: mFI-0)	−1.26 [−9.61, 7.10]	0.768		
mFI-2+ (ref: mFI-0)	−3.69 [−12.62, 5.24]	0.417		
Treatment duration (months)	−0.42 [−0.65, −0.19]	<0.001	−0.30 [−0.53, −0.07]	0.007
Age	−0.26 [−0.59, −0.07]	0.118	−0.39 [−0.72, −0.07]	0.005
BMI	−0.39 [−0.94, 0.16]	0.166	−0.35 [−0.89, 0.19]	0.098
HU pre-treatment	−0.18 [−0.2, −0.09]	<0.001	−0.19 [−0.28, −0.10]	<0.001
RAI	−0.08 [−0.40, 0.23]	0.607		
Active smoker	4.12 [−8.11, 16.36]	0.507		
Denosumab (ref: alendronate)	1.63 [−6.22, 9.48]	0.683		
Teriparatide (ref: alendronate)	20.09 [7.96,32.21]	0.001		
Romosozumab (ref: alendronate)	19.39 [7.72, 31.05]	0.001		

## Data Availability

Data are available upon request from the corresponding author.

## Supplementary Materials

The following supporting information can be downloaded at: https://www.mdpi.com/article/10.3390/jcm14093247/s1, Figure S1: Time interval of pre- and post-treatment CT.

## Abbreviations

BMD	bone mineral density
BMI	body mass index
CI	confidence interval
CT	computed tomography
DEXA	dual-energy X-ray absorptiometry
HR	hazard ratio
HU	Hounsfield unit
mFI5	5-item modified Frailty Index
mo	month
MR	magnetic resonance
PLIF	posterior lumbar interbody fusion
RAI	risk analysis index
VF	vertebral fracture
yr	year
PJK	proximal junctional kyphosis
