# Supplementary figures and images for: Impact of Frailty and Other Factors as Estimated by HU to Predict Response to Anabolic Bone Medications
Source: J Clin Med. 2025 May 7;14(9):3247. doi: 10.3390/jcm14093247 (PMC12072346; doi:10.3390/jcm14093247)

Patient

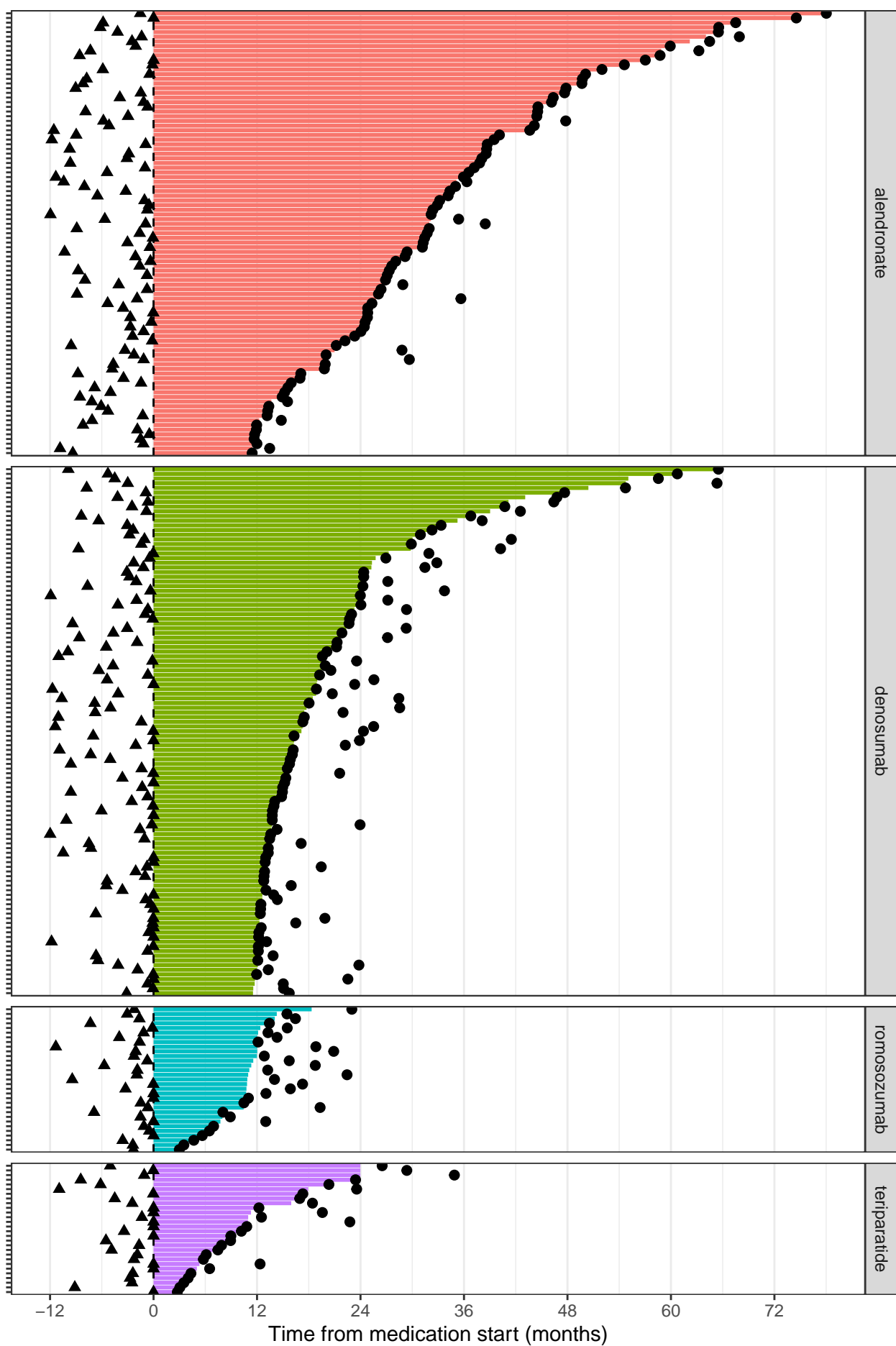

Supplement: Supplementary file 1 [file jcm-14-03247-s001.zip › jcm-3567089-Figure S1.pdf]
